# Annual of the Universal Medical Sciences

**Published:** 1888-09

**Authors:** 


**Annual of the Universal Medical Sciences.** A yearly report of the progress of the general sanitary sciences throughout the world. Edited by CHARLES E. SAJOUS, M. D., Lecturer on Laryngology and Rhinology in Jefferson Medical College, Philadelphia, etc., and seventy associate editors, assisted by over 200 corresponding editors, collaborators, and correspondents. Illustrated. Volumes I., II., III., IV. and V. 1888. Philadelphia and London: F. A. Davis, Publisher.

The impression conveyed by a first hasty glance at these volumes is one of favor; this develops into approbation and positive delight upon a more careful examination of the work. Its object, as stated in the editor's preface, is to collate the progressive features of medical literature, not only from sources of an accessible nature, but from those centers where no literature exists and clinical data are meagre. To this end medical journals, monographs, and treatises are laid under contribution, from which references, extracts, abstracts, and extended notices are made, supplemented in many instances by the individual experiences and opinions of the editors. The scope and import of the work can only be appreciated by its examination, but the following synopsis of its contents, given in the order of sequence as the subjects occur in the several volumes, will serve to exhibit its comprehensive nature:

Vol. I., pp. 15–541, comprises diseases of the brain and spinal cord; peripheral nervous diseases and general neuroses; diseases of the heart and pericardium; fevers; diseases of the mouth, stomach, pancreas, and liver; diseases of the intestines and peritoneum; animal parasites; diseases of the blood and spleen; tuberculosis and scrofula; rheumatism and gout; diabetes and diseases of the suprarenal capsules; diseases of the kidneys and bladder; chyluria and hæmoglobinuria; psychological diseases.

Vol. II., pp. 550, includes surgery of the brain and nerves; surgery of the abdomen; diseases of the rectum and anus; genito-urinary surgery; diseases and injuries of the art-ries and veins; fractures, dislocations, and sprains; diseases of the bones and joints; amputations, exsections, etc.; gunshot and punctured wounds; surgical tuberculosis, abscess, etc.; diseases of the skin; tumors; venereal diseases; surgical diseases; anæsthetics; surgical diagnosis.

Vol. III., pp. 563, treats of diseases of the eye; diseases of the ear; diseases of the nose and accessory cavities; diseases of the pharynx; diseases of the larynx, trachea and œsophagus; diseases of the thyroid gland; diseases of the lungs and plura in adults; inebriety, morphinism, and kindred diseases; oral surgery; dental pathology and therapeutics; prosthetic dentistry and orthodontia; surgical dressings; chiropodistry.

Vol. IV., pp. 548, discourses upon diseases of the uterus; menstruation and its disorders; diseases of the ovaries and tubes; diseases of the vagina and external genitals; diseases of pregnancy; obstetrics; puerperal diseases; dietetics of infancy and childhood; orthopedic surgery; general therapeutics; experimental therapeutics.

Finally, Vol. V., pp. 513, takes up climatology and balneology; electro therapeutics; medical chemistry and toxicology; legal medicine; medical demography; hygiene; disposal of the dead; anatomy of the brain; general anatomy; physiology; growth and age; technology; histology; embryology, anomalies, etc.; dental histology; general pathology.

As a specimen of medical book-making, it is altogether unique, nothing like it having yet appeared in any country. It should find a place on the shelves of the specialist, teacher, general practitioner, and theorist; the surgeon, statistician, sanitarian, and dentist. These and many others who either study, practice, or love the guild of medicine and the collateral sciences will find it of inestimable value. The three-column index furnishes a most complete and convenient form of finding the titles, therapeusis, and authors. The publisher deserves his full meed of praise for the beautiful and substantial make-up of the volumes.

